# Prognostic significance of platelet-to-albumin ratio in patients with esophageal squamous cell carcinoma receiving definitive radiotherapy

**DOI:** 10.1038/s41598-022-07546-0

**Published:** 2022-03-03

**Authors:** Zhiyu Huang, Qunhao Zheng, Yilin Yu, Hongying Zheng, Yahua Wu, Zhiping Wang, Lingyun Liu, Mengyan Zhang, Tianxiu Liu, Hui Li, Jiancheng Li

**Affiliations:** Fujian Cancer Hospital, Fujian Medical University Cancer Hospital, Fuzhou, 350014 China

**Keywords:** Cancer, Biomarkers

## Abstract

Accumulating evidence indicates that inflammation and nutrition status are associated with clinical outcomes in patients with various malignancies. This study aimed to evaluate the prognostic significance of the pretreatment platelet to albumin ratio (PAR) in esophageal squamous cell carcinoma (ESCC) patients undergoing definitive radiotherapy. A total of 470 patients who underwent definitive radiotherapy with or without chemotherapy were enrolled. The optimal cut-off values of PAR and other indicators were determined by the X-tile. The Kaplan–Meier method, multivariate analyses Cox regression were conducted to identify the association between those indicators and the survival outcomes. The median follow-up time was 23.5 months. The optimal cut-off value of PAR was 5.7 × 10^9^ and patients were stratified as the low PAR group and the high PAR group. In the univariate analysis, a low overall survival rate was significantly associated with T stage (*P* = 0.005), TNM stage (*P* < 0.001), Adjuvant chemotherapy (*P* = 0.007), neutrophil to lymphocyte ratio (NLR) (*P* = 0.006), platelet to lymphocyte ratio (*P* < 0.001), systemic immune-inflammation index (*P* < 0.001), prognostic nutritional index (*P* < 0.001) and platelet to albumin ratio (PAR) (*P* < 0.001). Patients with high PAR were associated with poorer OS and PFS than patients with low PAR. On multivariate analysis, TNM stage (*P* = 0.001), adjuvant chemotherapy (*P* < 0.001), and PAR (*P* = 0.033) were independent prognostic factors in ESCC treated with definitive radiotherapy. PAR is a novel, convenient, and inexpensive prognostic indicator for patients with ESCC undergoing definitive radiotherapy. Future validation from prospective larger-scale studies is warranted.

## Introduction

Esophageal cancer (EC) is one of the fatal tumor types throughout the world^1^. In China, EC is still a severe public health problem with high morbidity and mortality^2^. The majority of patients are diagnosed at advanced stages and lose the probability of curative resection. Despite the fact that alternative treatments such as immunotherapy and molecular-targeted therapy have been booming over the last decade, the clinical outcomes remain unsatisfactory^1,3^. Therefore, the identification of reliable indicators for prognosis prediction and individualized risk stratification has become a trending topic in the practice of EC research.

Accumulating evidence has revealed that nutritional status, cancer-related inflammation and the immune system play a vital role in carcinogenesis, proliferation, progression, and metastasis^4,5^. Several inflammation-based prognostic indicators such as the neutrophil to lymphocyte ratio (NLR), platelet to lymphocyte ratio (PLR), and systemic immune-inflammation index (SII) were demonstrated as independent prognostic factors in various solid tumors, including esophageal cancer, lung cancer, gastric cancer, colorectal cancer, and prostate cancer^6–10^. Several nutrition-based indicators including the prognostic nutritional index (PNI) are also known as critical prognostic elements in cancer patients. Published investigations have suggested a poor nutritional status was associated with a worse prognosis in various malignancies^11–13^. PAR comprises both platelet count and serum albumin concentration and is applied to assess the inflammation-nutrition status. Several studies have reported that PAR could serve as independent prognostic markers in some types of cancers, including lung cancer^14^, hepatocellular cancer^15^, Pancreatic cancer^16^, and cholangiocarcinoma^17^. However, the relationship between the pretreatment PAR and the survival of patients with esophageal squamous cell carcinoma (ESCC) remains uncertain.

To the best of our knowledge, the prognostic role of PAR in ESCC patients who received definitive radiotherapy has not been reported. Therefore, in the current study, we aimed to evaluate whether the pretreatment PAR was associated with the clinical outcomes and could serve as an independent prognostic factor in ESCC patients undergoing definitive radiotherapy.

## Materials and methods

### Patients

Patients who underwent definitive radiotherapy for esophageal cancer were retrospectively reviewed from January 2011 to December 2015 at our center. The inclusive criteria were as follows: (I) histologically confirmed as ESCC; (II) Karnofsky score ≥ 70; (III) treated with definitive radiotherapy (50–70 Gy in 25–35 fractions, 5 days per week) with 0–6 cycles of platinum-based chemotherapy; (IV) complete clinicopathological and pretreatment serum laboratory data; (V) neither the history of malignancy nor acute and/or chronic inflammatory disease and (VI) restaged according to the 8th edition of the American Joint Committee on Cancer TNM staging system for esophageal cancer^18^. The study was approved by the the Ethics Committee of the Fujian Medical University Cancer Hospital. Written informed consent was waived by the the Ethics Committee of the Fujian Medical University Cancer Hospital due to the retrospective nature of the study. All methods were performed in accordance with relevant guidelines and regulations (the ethical standards of the 1964 Declaration of Helsinki and its later amendments or comparable ethical standards).

### Treatment protocol

Radiotherapy was delivered by two-dimensional conventional radiotherapy (2D-CRT), three-dimensional conformal radiation therapy (3D-CRT), or intensity-modulated radiation therapy (IMRT) technique in this study. The gross tumor volume (GTV) was determined by the contrast-enhanced CT, barium swallow, endoscopic examination, or PET-CT, which contained both the primary tumor and the positive regional lymph nodes. The clinical target volume (CTV) is composed of subclinical lesions (GTV extending 0.5–1.0 cm in axial direction and 3 cm in longitudinal direction) and the relative mediastinal lymphatic drainage field. The CTV plus a 0.5 cm expansion margin in all directions was defined as the planning target volume (PTV). Patients who received two-dimensional conventional radiotherapy (2DRT) used anterior and posterior opposing techniques. The irradiated field included the primary tumor and a distal and proximal margin of 3 cm and a 0.5–1.0 cm radial margin around the tumor.

The Platinum-based two-drug chemotherapy regimen was used for some enrolled patients: (I) paclitaxel 135 mg/m^2^ D1 or docetaxel 75 mg/m^2^ D1 + cisplatin or nedaplatin 75 mg/m^2^ D2; (II) 5-fluorouracil (5-FU) 700–1000 mg/m^2^ D1–2 + cisplatin 75 mg/m^2^ D2, every three weeks as a cycle.

### Definition of the NLR, PLR, SII, PNI and PAR

The baseline neutrophil, lymphocyte, platelet counts, and serum albumin levels were collected from routine test reports 7 days prior to the first treatment. The definitions of NLR, PLR, SII, PNI and PAR are calculated as follows:

NLR = absolute neutrophil count/absolute lymphocyte count;

PLR = platelet counts/absolute lymphocyte count;

SII = platelet counts* absolute neutrophil count/absolute lymphocyte count;

PNI = serum albumin level (g/L) + 5* absolute lymphocyte count;

PAR = platelet counts/serum albumin level (g/L).

The optimal cutoff values for the NLR, PLR, SII, PNI and PAR were determined by X-tile software (http://www.tissuearray.org/rimmlab)^19^.

### Follow-up

During treatment, patients were assessed weekly to screen the treatment toxicities, including blood routine, biochemical test and physical examination. After treatment, Follow-up examinations were conducted 1 month after finishing radiotherapy, and then every 3 months in the first year, every 6 months over the next 2 years, and once a year thereafter. The routine examination items included physical examination, laboratory tests, tumor markers, thoracic CT scanning, and esophageal barium. The followed up lasted until death or the last contact. The primary endpoint was overall survival (OS), which was defined as the period from treatment initiation to the date of last follow-up or death from any cause. Progression-free survival (PFS) was defined as the period from treatment initiation to the date of disease progression or death from any cause. Disease progression was evaluated by the standard Response Evaluation Criteria in Solid Tumors^20^.

### Statistical analysis

All Statistical analysis was performed with SPSS 26.0 (SPSS, Chicago, IL). The association between the PAR groups and clinicopathological characteristics was analyzed by the χ^2^ test. Survival curves for OS and PFS were plotted via the Kaplan–Meier method and compared by the log-rank test. Univariate and multivariate cox analyses were conducted to identify the independent risk or prognostic factors. A *P* value < 0.05 was considered as statistically significant.

## Result

### Patient characteristics

The baseline clinicopathological characteristics of 470 patients in the study were shown in Table 1. All patients consisted of 333 (70.9%) men and 137 (29.1%) women, with the median age of 64 years (range: 36–94 years). 43 (9.1%), 109 (23.2%), 271 (57.7%), and 47 (10.0%) patients had primary tumors located in the cervical, upper, middle, and lower thoracic esophagus respectively. Based on the criteria of the 8th edition AJCC TNM staging system, 95 (20.2%), 142 (30.2%), and 233 (49.6%) of cases were diagnosed as stage II, stage III, and stage IV. Thirty-two patients received 2D-CRT, and 438 patients received 3D-CRT and IMRT. Besides, there were 321 (68.3%) patients who received adjuvant chemotherapy.

**Table 1:** Baseline clinical variables of the study participants stratified by pretreatment PAR.

Variables	Total, n(%)	Low PAR (N = 172)	High PAR (N = 298)	*P*-value
**Gender**				0.007
Male	333 (70.9)	109	224	
Female	137 (29.1)	63	74	
**Age**				0.020
≤ 70	305 (64.9)	100	205	
> 70	165 (35.1)	72	93	
**Location**				0.124
Cervical	43 (9.1)	22	21	
Upper third	109 (23.2)	41	68	
Middle third	271 (57.7)	96	175	
Lower third	47 (10.0)	13	34	
**T stage**				0.003
T2	43 (9.1)	19	24	
T3	217 (46.2)	94	123	
T4	210 (44.7)	59	151	
**N stage**				0.084
N0	118	51	67	
N +	352	121	231	
**cTNM stage**				0.001
II	95 (20.2)	46	49	
III	142 (30.2)	59	83	
IV	233 (49.6)	67	166	
**RT technique**				0.158
2D-CRT	32 (6.8)	8	24	
IMRT + 3D-CRT	438 (93.2)	164	274	
**Chemotherapy**				0.031
No	149 (31.7)	65	84	
Yes	321 (68.3)	107	214	
**Marrow depression**				0.568
No	224 (47.7)	79	145	
Yes	246 (52.3)	93	153	
**NLR**				0.297
< 2.62	275 (58.5)	106	169	
≥ 2.62	195 (41.5)	66	129	
**PLR**				< 0.001
< 180	364 (77.4)	156	208	
≥ 180	106 (22.6)	16	90	
**SII**				< 0.001
< 577.7	254 (54.0)	131	123	
≥ 577.7	216 (46.0)	41	175	
**PNI**				0.499
< 41.5	64 (13.6)	21	43	
≥ 41.5	406(86.4)	151	255	

*PAR* platelet to albumin ratio, *NLR* neutrophil to lymphocyte ratio, *PLR* platelet to lymphocyte ratio, *SII* systemic immune-inflammation index, *PNI* prognostic nutritional index, *2DRT* two-dimensional conformal radiation therapy, *3DRT* three-dimensional conformal radiation therapy, *IMRT* intensity-modulated radiation therapy.

The optimal cutoff values for the NLR, PLR, SII, PNI and PAR calculated by the X-tile was 2.62, 180, 577.7, 41.5, and 5.7 × 10^9^, respectively.(Supplementary Figs. 1 and 2) Then patients were divided into the low PAR group (PAR < 5.7 × 10^9^) and the high PAR group (PAR ≥ 5.7 × 10^9^) for further analyses.

### Association between PAR and clinicopathological characteristics

**Figure. 1:**
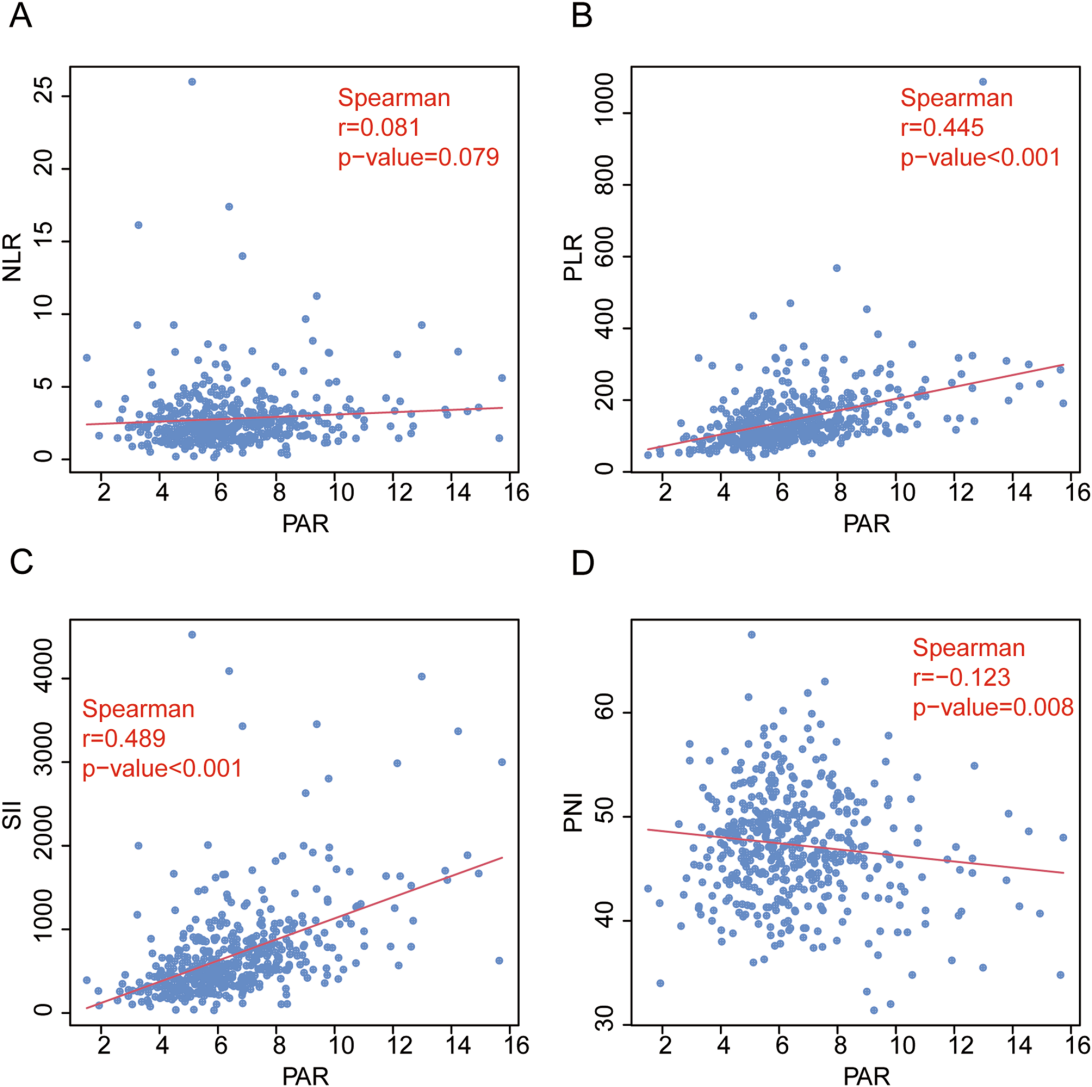
Correlations between PAR and (**A**) NLR, (**B**) PLR, (**C**) SII and (**D**) PNI were evaluated via Spearman’s correlation analysis in the whole patients.

The association between the PAR and the patient clinicopathological variables of this study are shown in Table 1. We found that high PAR level was significantly associated with male (*p* = 0.007), age  ≤ 70 years old (*p* = 0.020), deeper tumor invasion (T stage, *p* = 0.003), more advanced TNM stage *(p* = 0.001), Chemotherapy (*p* = 0.031), higher PLR (*p* < 0.001) and higher SII (*p* < 0.001). In additional, Spearman analyses showed a positive correlation between PAR and PLR (r = 0.445, *P* < 0.001; Fig. 1B) and SII (r = 0.489, *P* < 0.001; Fig. 1C). The PAR was negatively correlated with the PNI (r =  − 0.123, *P* = 0.008; Fig. 1D) and no significant correlation with NLR (r = 0.081, *P* = 0.079; Fig. 1A).

### The prognostic significance of NLR, PLR SII, PNI and PAR

**Figure. 2:**
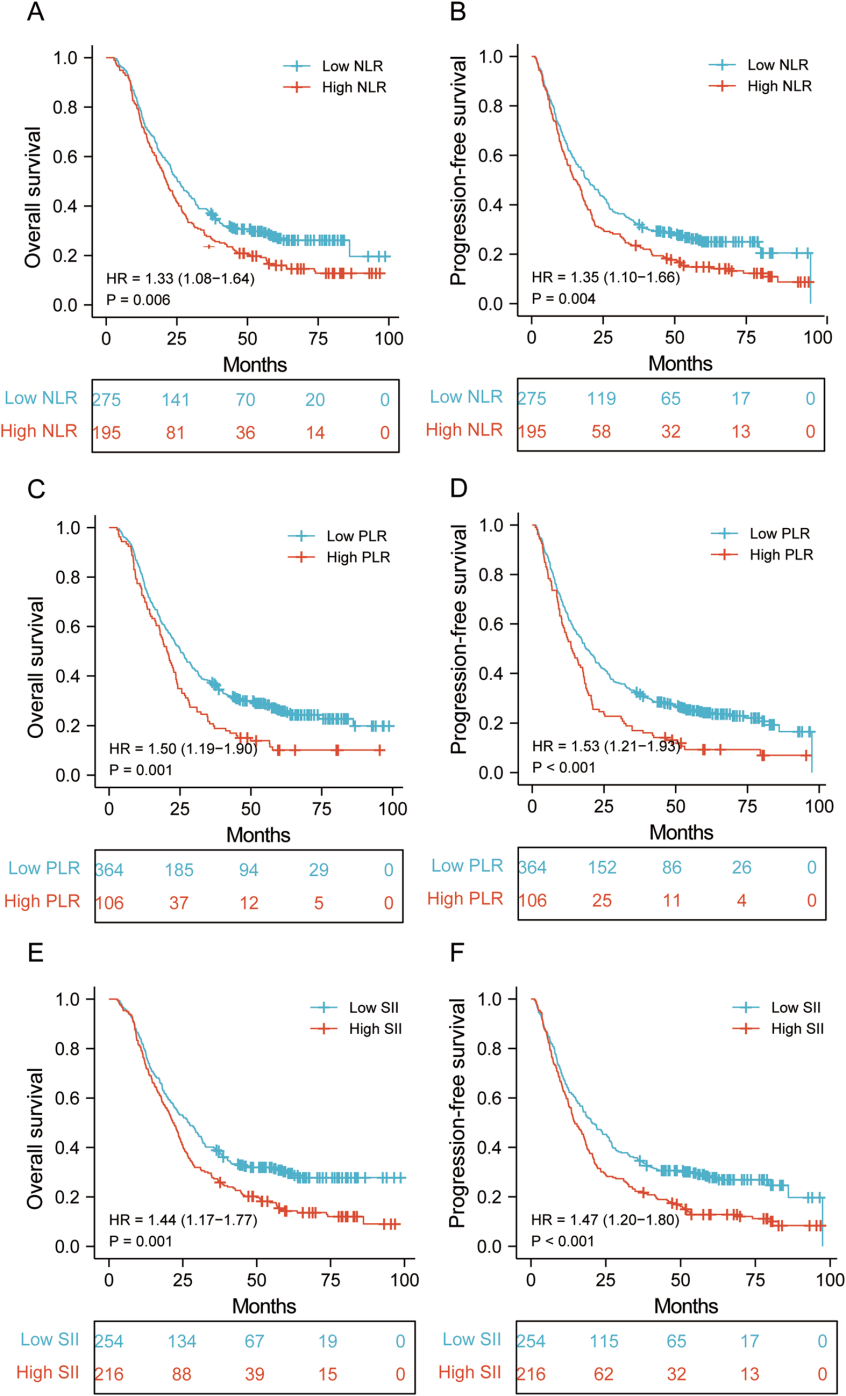
Survival outcomes in ESCC patients undergoing definitive radiotherapy stratified by NLR, PLR and SII. (**A**) Overall survival and (**B**) progression-free survival between low and high NLR groups; (**C**) Overall survival and (**D**) progression-free survival between low and high PLR groups. (**E**) Overall survival and (**F**) progression-free survival between low and high SII groups.

**Figure. 3:**
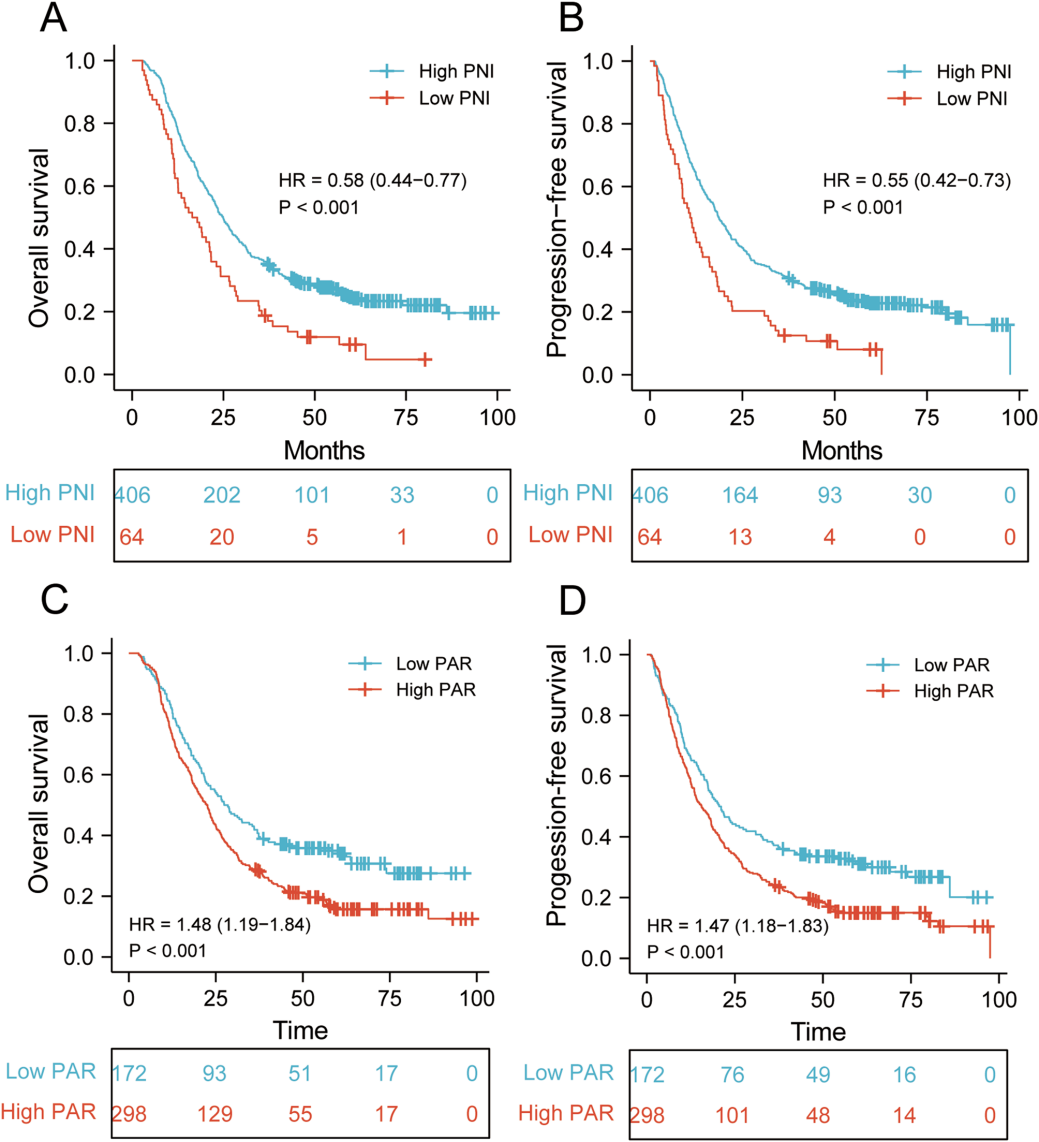
Survival outcomes in ESCC patients undergoing definitive radiotherapy stratified by PNI, PAR. (**A**) Overall survival and (**B**) progression-free survival between low and high PNI groups; (**C**) Overall survival and (**D**) progression-free survival between low and high PAR groups.

The median follow-up time was 23.5 months (range 2–98.7 months). The Kaplan–Meier survival analysis demonstrated that higher NLR, PLR, SII, and PAR were significantly correlated with shorter survival time (Figs. 2 and 3). The 1-, 3-, and 5-year PFS of ESCC patients with low and high PARs were 66.9%, 37.2%, 31.1%; and 59.7%, 24.2%, 15.0%, respectively (Fig. 3D). The low-PAR group had a significantly higher PFS rate than that of the high-PAR group (*P* < 0.001). The 1-, 3-, and 5-year OS rates were 74.5%, 28.9%, and 15.7%, respectively, in the high-PAR group and 83.1%, 42.4%, and 35.0%, respectively, in the low-PAR group (Fig. 3C).

Univariate Cox regression analysis demonstrated T stage, TNM stage, adjuvant chemotherapy, NLR, PLR, SII, PNI and PAR prognostic factors (*P* = 0.005, *P* < 0.001, *P* = 0.003, *P* = 0.004,* P* < 0.001, *P* < 0.001, *P* < 0.001, and *P* < 0.001, respectively for PFS; *P* = 0.005, *P* < 0.001, *P* = 0.007, *P* = 0.006, *P* < 0.001, *P* < 0.001, *P* < 0.001 and *P* < 0.001,respectively for OS) and as significant prognostic factor for both PFS and OS (Tables 2 and 3). All the eight clinicopathological characteristics were further investigated in the multivariate cox regression analysis. As shown in Table 2, TNM stage [hazard ratio (HR) 2.147, 95% confidence interval (CI): 1.376–3.351, *P* < 0.001], adjuvant chemotherapy (HR: 0.618, 95% CI: 0.493–0.774, P < 0.001), PNI (HR: 0.691, 95% CI: 0.505–0.944, *P* = 0.020) and PAR (HR: 1.298, 95% CI: 1.014–1.661, *P* = 0.038) were identified as independent prognostic factors in ESCC patient for predicting PFS. And in Table 3, TNM stage (HR: 2.147, 95% CI: 1.350–3.414, *P* = 0.001), adjuvant chemotherapy (HR: 0.647, 95% CI: 0.515–0.813, *P* < 0.001) and PAR (HR: 1.312, 95% CI: 1.021–1.685, *P* = 0.033) were identified as independent prognostic factors in ESCC patient for predicting OS.

**Table 2:** Univariable and multivariable Cox regression analysis for progression-free survival.

Variables	Univariate analysis		Multivariate analysis	
	HR (95% CI)	*P* value	HR (95% CI)	*P* value
**Gender**				
Male/Female	0.983 (0.784–1.232)	0.879		
**Age**				
≤ 70/ > 70	1.046 (0.846–1.292)	0.679		
**Location**				
Cervical + Upper/Middle + Lower	1.155 (0.926–1.440)	0.202		
**T stage**				
T2-3/T4	1.335 (1.089–1.636)	0.005	0.683 (0.439–1.063)	0.091
**N stage**				
N0/N +	1.240 (0.973–1.581)	0.083		
**cTNM stage**				
II + III/IV	1.458 (1.188–1.788)	< 0.001	2.147 (1.376–3.351)	< 0.001
**RT technique**				
2D-CRT/IMRT + 3D-CRT	0.877 (0.593–1.298)	0.512		
**Adjuvant chemotherapy**				
No/Yes	0.726 (0.586–0.898)	0.003	0.618 (0.493–0.774)	< 0.001
**Marrow depression**				
No/Yes	0.820 (0.670–1.005)	0.055		
**NLR**				
< 2.62/ ≥ 2.62	1.352 (1.103–1.658)	0.004	1.103 (0.823–1.479)	0.513
**PLR**				
< 180/ ≥ 180	1.529 (1.211–1.930)	< 0.001	1.109 (0.833–1.476)	0.478
**SII**				
< 577.7/ ≥ 577.7	1.466 (1.196–1.796)	< 0.001	1.209 (0.886–1.649)	0.232
**PNI**				
< 41.5/ ≥ 41.5	0.554 (0.419–0.733)	< 0.001	0.691 (0.505–0.944)	0.020
**PAR**				
< 5.7 × 10^9^/ ≥ 5.7 × 10^9^	1.470 (1.183–1.827)	< 0.001	1.298 (1.014–1.661)	0.038

*HR* hazard ratio, *CI* confidence interval.

**Table 3:** Univariable and multivariable Cox regression analysis for overall survival.

Variables	Univariate analysis		Multivariate analysis	
	HR (95% CI)	*P* value	HR (95% CI)	*P* value
**Gender**				
Male/Female	0.994 (0.790–1.250)	0.960		
**Age**				
≤ 70/ > 70	1.008 (0.812–1.251)	0.944		
**Location**				
Cervical + Upper/Middle + Lower	1.086 (0.870–1.356)	0.468		
**T stage**				
T2-3/T4	1.341 (1.091–1.648)	0.005	0.667 (0.421–1.057)	0.085
**N stage**				
N0/N +	1.223 (0.957–1.562)	0.107		
**cTNM stage**				
II + III/IV	1.461 (1.188–1.798)	< 0.001	2.147 (1.350–3.414)	0.001
**RT technique**				
2D-CRT/IMRT + 3D-CRT	0.769 (0.516–1.147)	0.199		
**Adjuvant chemotherapy**				
No/Yes	0.742 (0.597–0.923)	0.007	0.647 (0.515–0.813)	< 0.001
**Marrow depression**				
No/Yes	0.861 (0.701–1.057)	0.152		
**NLR**				
< 2.62/ ≥ 2.62	1.333 (1.085–1.639)	0.006	1.108 (0.820–1.496)	0.505
**PLR**				
< 180/ ≥ 180	1.503 (1.187–1.902)	< 0.001	1.095 (0.817–1.467)	0.545
**SII**				
< 577.7/ ≥ 577.7	1.440 (1.172–1.769)	< 0.001	1.170 (0.852–1.606)	0.333
**PNI**				
< 41.5/ ≥ 41.5	0.581 (0.438–0.770)	< 0.001	0.732 (0.532–1.007)	0.055
**PAR**				
< 5.7 × 10^9^/ ≥ 5.7 × 10^9^	1.480 (1.187–1.845)	< 0.001	1.312 (1.021–1.685)	0.033

*HR* hazard ratio, *CI* confidence interval.

**Figure. 4:**
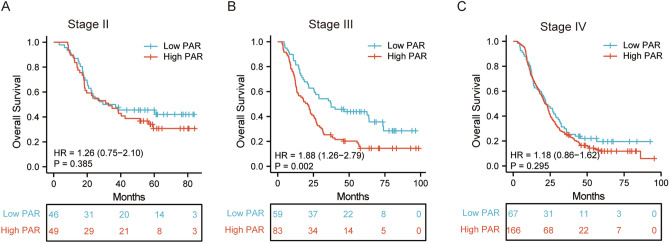
Survival outcomes in ESCC patients undergoing definitive radiotherapy stratified by PAR in stage II (**A**), stage III (**B**), and stage IV (**C**) subgroup.

Furthermore, we explored the prognostic value of PAR in a subgroup analysis which was stratified by AJCC TNM stage. The result showed the high-PAR was significant shorter OS in stages III (*P* = 0.002), Fig. 4B). Although there was no statistical significance in the subgroup analyses of OS in stages II (*P* = 0.385, Fig. 4A) and stage IV (*P* = 0.295, Fig. 4C), the trend of worse prognosis in high-PAR patients was consistent.

## Discussion

Inflammation in the microenvironment of tumors is known to promote both carcinogenesis and disease progression and could reduce the efficacy of systemic treatments^21^. Platelet, as a critical component in hemostasis, is also observed to be associated with systemic inflammation and the immune system^22,23^. Nutritional status is also a critical part of cancer management as malnutrition is frequent in cancer patients and is correlated with poor prognosis^24–26^. Previous studies have demonstrated albumin-related malnutrition was significantly associated with poor prognosis in ESCC patients^27,28^. Hence, a novel inflammation-based indicator PAR was established, representing both inflammation and nutritional status.

The current study first evaluated the prognostic value of a pretreatment PAR in ESCC patients who received definitive radiotherapy ± adjuvant chemotherapy. Our results showed that patients with a low pretreatment PAR(< 5.7 × 10^9^) had a significantly better prognosis in both PFS and OS than those with a high pretreatment PAR(≥ 5.7 × 10^9^). A high PAR was more likely to associate with an younger age, a more advanced TNM stage, suggesting that PAR could reflect tumor progression in patients with ESCC. Multivariable analyses also validated the PAR as an independent indicator of PFS and OS. Taken together, our results demonstrated that pretreatment PAR is an independent prognostic factor for patients with ESCC who received definitive radiotherapy.

Comparing to PAR, the existing inflammation-based indicators, including NLR, PLR, SII and PNI, showed no statistical significance as an independent prognostic factor in the multivariable cox regression analysis. Previous studies showed that high pretreatment of NLR and PLR were independent prognostic markers for OS in esophageal cancer patients. In the current study, NLR and PLR showed a correlation with OS but failed to maintain significance in multivariable cox analysis. The findings were consistent with Geng et al. research^29^. Likewise, SII also demonstrated no independent prognostic value in this study.

It’s been known that inflammation not only participates in tumorigenesis, malignant progression, and metastasis but also interplay with antitumor immunity^30^. A number of studies have revealed that high platelet counts can affect tumor development and result in thrombocytosis, which is an unfavorable factor for patients’ clinical outcomes^31^. It has been hypothesized that platelet was activated by cytokines secreted by tumor cells, such as platelet-derived growth factor (PDGF), vascular endothelial growth factor (VEGF), transforming growth factor-β1 (TGF-β1). Those cytokines are components of platelet and play a critical role in tumor development, including tumor proliferation, angiogenesis, and metastasis^32^. Moreover, platelet could shield peripheral circulating tumor cells and interfere with natural killer cells for recognition of tumor cells, which enhanced their metastatic potential^33^. Most recently, more emphasis has been put on the platelets and their complicated interplay with leukocytes and tumor cells in the tumor microenvironment, which could help us to better understand the mechanism behind the commonly used antiplatelet therapy and shed light on novel cancer therapy ^34^.

Low serum albumin concentration could also lead to a high PAR. Previous studies have demonstrated that pretreatment serum albumin is associated with short life expectancy in cancer patients^35^. Malnutrition is a significant problem in digestive cancer patients, especially in those with locally advanced esophageal cancer undergoing definitive radiotherapy. The tumor-caused restriction of oral intake and radiation-induced esophagitis are the main reasons that account for undernutrition. Patients with an undernutrition status before treatment were demonstrated to be associated with poor treatment response and prognosis^36^. In our study, we found that patients with high serum albumin concentration had a better survival outcome than those with low serum albumin concentration (as shown in Supplementary Fig. 3), which is consistent with previous findings. Moreover, albumin synthesis was suppressed by the systemic inflammation. Consequentially the immune system functions were impaired due to hypoalbuminemia, and tumor cells could progress more easily due to immune suppression. Therefore, risk stratification based on inflammation-nutritional indicators is of great significance and will help the clinical physician to provide timely and effective nutritional intervention.

Although our study demonstrated the prognostic significance of PAR in patients with ESCC, several limitations in our study still need to be noted. First, this is a retrospective study from a single center, which may lead to selection bias. Second, platelet counts and serum albumin levels could be influenced by other factors such as coagulation disorder and liver dysfunction, which might affect the predictive accuracy of prognosis. Third, the optimal cut-off point of PAR might fluctuate as the evaluated population changed. Hence, further larger-scale prospective studies are needed to confirm the preliminary results of our study.

## Conclusion

Our study demonstrated that the PAR, a novel independent risk predictor, had the potential for predicting prognosis of ESCC patients undergoing definitive radiotherapy. Measurement of the PAR is convenient, inexpensive, and reliable in the routine clinical practice. Anti-inflammation therapy and/or nutritional interventions should be considered for patients with low pretreatment PAR levels. Therefore, PAR measurement will help the clinical decision-making according to the individual difference. Future validation from prospective larger-scale studies is warranted.

## Supplementary Information

Supplementary Legends.

Supplementary Figure 1.

Supplementary Figure 2.

Supplementary Figure 3.

## Data Availability

The datasets used and/or analyzed during the current study are available from the corresponding author on reasonable request.

## Abbreviations

PAR: Platelet to albumin ratio
ESCC: Esophageal squamous cell carcinoma
PFS: Progression-free survival
OS: Overall survival
EC: Esophageal cancer
PNI: Prognostic nutritional index
2DRT: Two-dimensional conformal radiation therapy
3DRT: Three-dimensional conformal radiation therapy
IMRT: Intensity-modulated radiation therapy
NLR: Neutrophil to lymphocyte ratio
PLR: Platelet to lymphocyte ratio
SII: Systemic immune-inflammation index
GTV: Gross tumor volume
CTV: Clinical target volume
PTV: Planning target volume
