# Supplementary material for: Prognostic significance of platelet-to-albumin ratio in patients with esophageal squamous cell carcinoma receiving definitive radiotherapy
Source: Sci Rep. 2022 Mar 3;12:3535. doi: 10.1038/s41598-022-07546-0 (PMC8894409; doi:10.1038/s41598-022-07546-0)
Supplement: Supplementary file 1 — Supplementary Legends. [file 41598_2022_7546_MOESM1_ESM.docx]

**Supplementary Figure 1** X-tile analyses. The optimal cut-off value for NLR, PLR, SII were 2.62 (A),180 (B) and 577.7 (C) according to the X-tile program.

Abbreviations: NLR, neutrophil to lymphocyte ratio; PLR, platelet to lymphocyte ratio; SII, systemic immune-inflammation index. NLR, neutrophil to lymphocyte ratio; PLR, platelet to lymphocyte ratio; SII, systemic immune-inflammation index.

**Supplementary Figure 2** X-tile analyses. The optimal cut-off value for PNI and PAR were 41.5 (A), 5.7 (B) according to the X-tile program.

Abbreviations: PAR, platelet to albumin ratio; PNI, prognostic nutritional index.

**Supplementary Figure 3** (A): The optimal cutoff values of albumin determined by X-tile software; (B): Overall survival and progression-free survival between low and high albumin groups.
