# Supplementary figures and images for: Prognostic significance of platelet-to-albumin ratio in patients with esophageal squamous cell carcinoma receiving definitive radiotherapy
Source: Sci Rep. 2022 Mar 3;12:3535. doi: 10.1038/s41598-022-07546-0 (PMC8894409; doi:10.1038/s41598-022-07546-0)

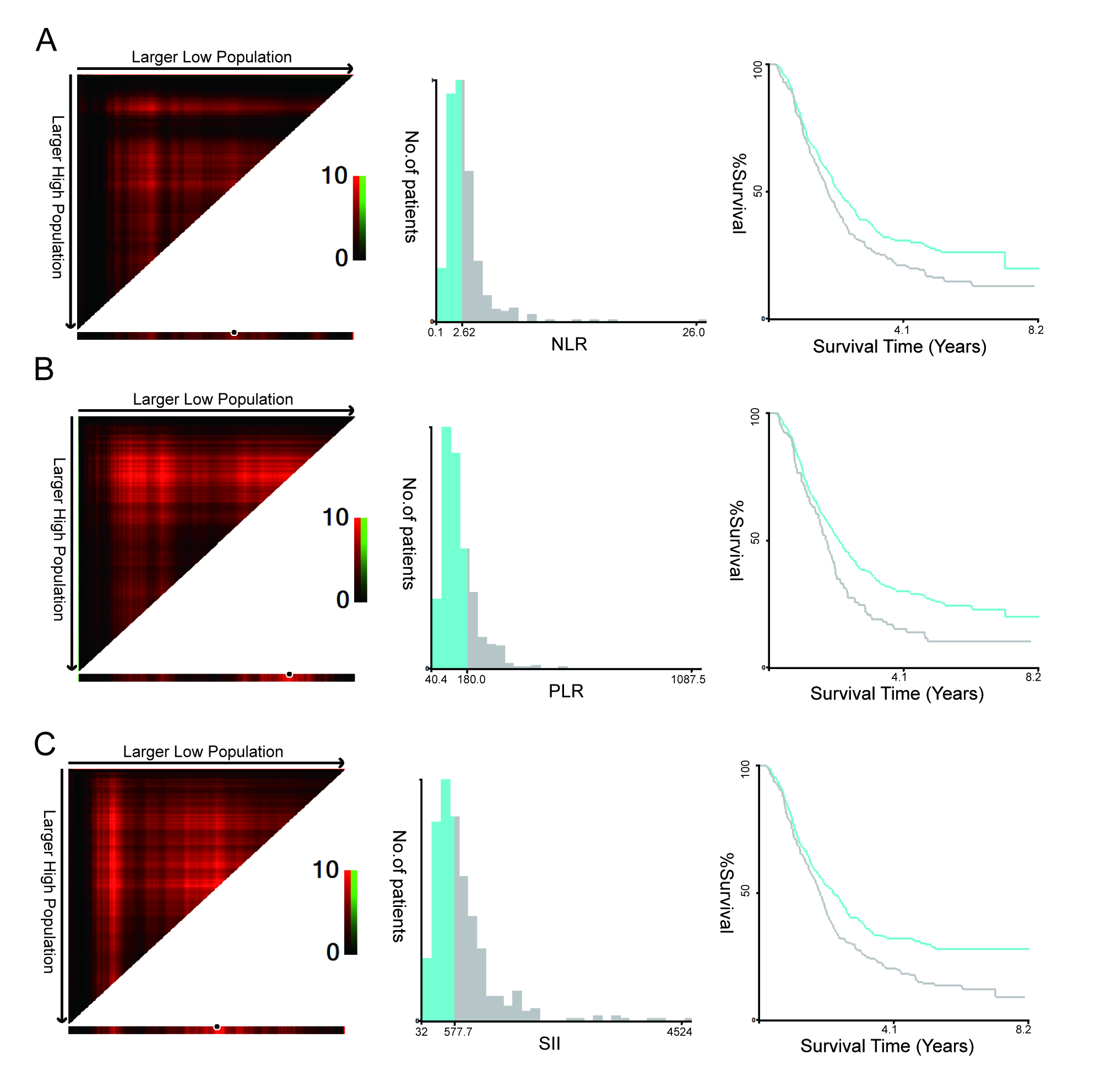

Supplement: Supplementary file 2 — Supplementary Figure 1. [file 41598_2022_7546_MOESM2_ESM.tif]

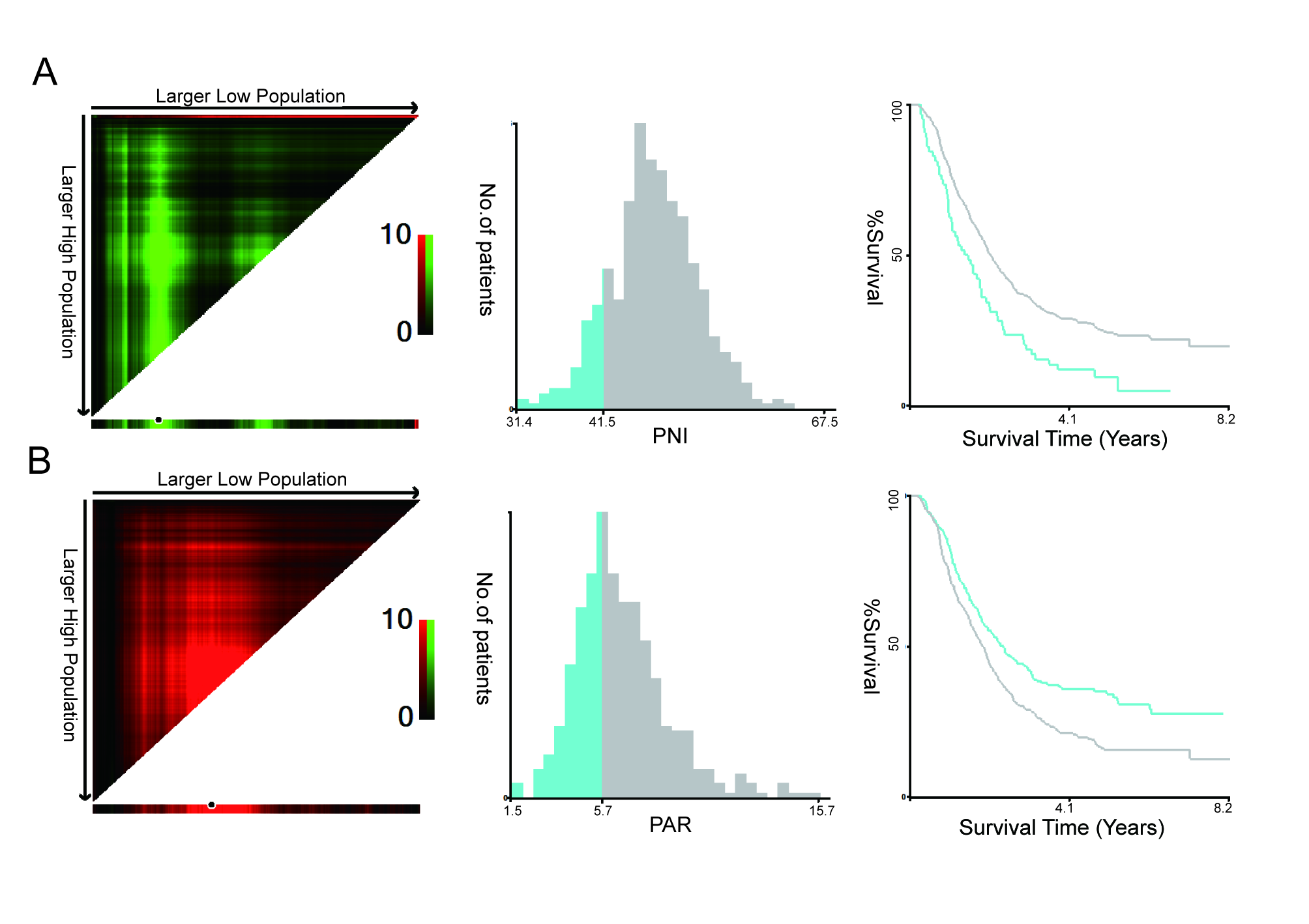

Supplement: Supplementary file 3 — Supplementary Figure 2. [file 41598_2022_7546_MOESM3_ESM.tif]

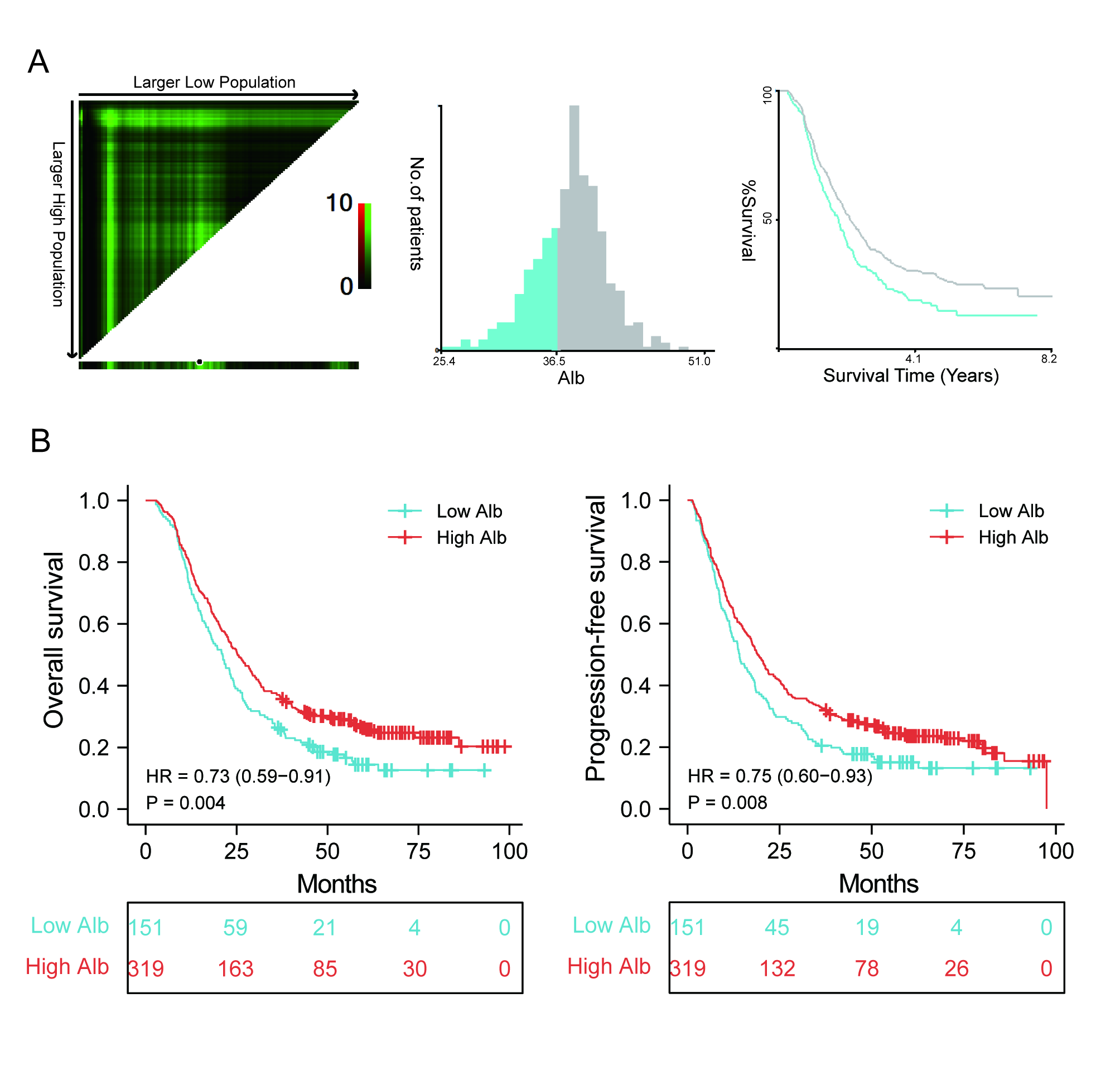

Supplement: Supplementary file 4 — Supplementary Figure 3. [file 41598_2022_7546_MOESM4_ESM.tif]
